# Capacitive Field-Effect Biosensor Studying Adsorption of *Tobacco Mosaic Virus* Particles

**DOI:** 10.3390/mi12010057

**Published:** 2021-01-06

**Authors:** Melanie Jablonski, Arshak Poghossian, Robin Severins, Michael Keusgen, Christina Wege, Michael J. Schöning

**Affiliations:** 1 Institute of Nano- and Biotechnologies, FH Aachen, 52428 Jülich, Germany; m.jablonski@fh-aachen.de (M.J.); robin.severins@alumni.fh-aachen.de (R.S.); 2 Institute of Pharmaceutical Chemistry, Philipps University Marburg, 35032 Marburg, Germany; Michael.keusgen@staff.uni-marburg.de; 3 MicroNanoBio, 40479 Düsseldorf, Germany; 4 Institute of Biomaterials and Biomolecular Systems, University of Stuttgart, 70569 Stuttgart, Germany; Christina.wege@bio.uni-stuttgart.de; 5 Institute of Biological Information Processing (IBI-3), Forschungszentrum Jülich, 52425 Jülich, Germany

**Keywords:** capacitive field-effect sensor, plant virus detection, *tobacco mosaic virus* (TMV), TMV adsorption, Ta_2_O_5_ gate, bioreceptor nanocarrier, streptavidin-penicillinase conjugates

## Abstract

Plant virus-like particles, and in particular, *tobacco mosaic virus* (TMV) particles, are increasingly being used in nano- and biotechnology as well as for biochemical sensing purposes as nanoscaffolds for the high-density immobilization of receptor molecules. The sensitive parameters of TMV-assisted biosensors depend, among others, on the density of adsorbed TMV particles on the sensor surface, which is affected by both the adsorption conditions and surface properties of the sensor. In this work, Ta_2_O_5_-gate field-effect capacitive sensors have been applied for the label-free electrical detection of TMV adsorption. The impact of the TMV concentration on both the sensor signal and the density of TMV particles adsorbed onto the Ta_2_O_5_-gate surface has been studied systematically by means of field-effect and scanning electron microscopy methods. In addition, the surface density of TMV particles loaded under different incubation times has been investigated. Finally, the field-effect sensor also demonstrates the label-free detection of penicillinase immobilization as model bioreceptor on TMV particles.

## 1. Introduction

Recently, exponential advances in nano- and biotechnological applications of viruses and virus-like particles have stimulated their implementation as templates for the synthesis and assembly of nanomaterials with diverse hierarchical structures and as sensing components in biochemical sensors [1,2,3,4,5,6,7,8]. In contrast to chemically synthesized particles, which are typically polydisperse, several types of biological virus nanoparticles have the unique advantage that they can be produced in a large number of identical copies as determined by their genetics. Especially, plant virus-like particles are increasingly being used for nanobiotechnology purposes due to their robustness, high surface-to-volume ratio, highly controllable and precisely defined structures, inherent biodegradability and biocompatibility, lack of toxicity and pathogenicity in humans and other mammals, and the possibility of low-cost production from infected plants in a large quantity [1,2,3,4,9,10,11,12,13,14,15].

The *tobacco mosaic virus* (TMV) is one of the most studied plant viruses and is formed by ca. 2130 identical copies of coat proteins helically assembled around a single-stranded genomic ribonucleic acid molecule [3,12,16,17,18,19]. The TMV represents a high-aspect ratio, rigid, nanotube-like biological nanoparticle with a length of 300 nm for complete viruses, an outer diameter of 18 nm and a longitudinal cylindrical internal channel of 4 nm diameter [18,19,20,21]. The TMV is able to infect numerous plants, many of them in the family *Solanaceae* (e.g., tobacco, tomato, bell pepper) causing characteristic mosaic-like dark- and light-green patterns on the leaves of infected tobacco, while it is completely harmless for mammals [1,22,23]. The TMV particles display excellent chemical and physical stability and can withstand temperatures up to 90 °C [14,15] and, under near-physiological conditions, pH values between ~3.0 and ~8.5 [22]. They can survive for years in cigars and cigarettes made from infected leaves [24]. TMV nanotubes exhibit an extremely high number of precisely positioned surface docking sites that can be used for the high-density immobilization of biomolecules (e.g., antibodies, antigens, enzymes) [1,9,25,26]. Finally, TMV particles as receptor nanocarriers can be easily integrated with various transducers, that is making them highly convenient for (bio-) chemical sensing applications. For instance, TMV scaffolds coated with palladium nanoparticles were utilized as sensing material to develop surface acoustic wave hydrogen sensors [7], while a voltammetric electrochemical sensor chip with TMV particles was proposed for detecting the explosive agent trinitrotoluene [27]. In addition, TMV particles were integrated with an optical microdisk resonator [28] and impedimetric sensor [29] for the label-free antibody detection. Moreover, TMV-assisted colorimetric glucose and penicillin biosensors were developed in [25,30].

Recently, we presented an amperometric glucose biosensor, where TMV particles were loaded onto the sensor surface and used as enzyme nanocarriers to immobilize glucose oxidase [26,31]. In further work, a TMV-modified penicillin field-effect biosensor with immobilized penicillinase (Pen) was introduced [32]. The sensor represents a capacitive EIS (electrolyte-insulator-semiconductor) structure. The working principle of this TMV/enzyme-modified EIS biosensor is based on the detection of pH changes near the EIS sensor surface resulting from the enzymatic reaction. These first successful experiments underline the potential to integrate TMV/bioreceptor hybrids with electronic transducers. Since field-effect sensors are able to directly convert specific (bio)molecular interactions into electrical signals, they have been widely applied such as for the detection of enzymatic reactions, cell acidification and cellular signals, DNA (deoxyribonucleic acid), antibody-antigen affinity binding, neurotransmitters, and viruses (see e.g., [33,34,35,36,37,38,39,40,41,42,43]). The functionalization of field-effect sensors with TMV nanocarriers enables a universal approach to engineer a large variety of biosensors and biochips.

Similar to other nanoparticle-modified biosensors [44,45,46], generally, the presence of TMV nanocarriers on the sensor surface can provide an increased receptor density, an enhanced diffusion transport of analyte molecules for nanoparticle surfaces in comparison to planar surfaces, and a favorable orientation and accessibility of receptors for target analytes to be detected (see, e.g., [13,31]), all resulting in an enhanced biosensor performance. The sensing characteristics (sensitivity, detection limit and concentration range, etc.) of these biosensors, among others, depend on the density of TMV particles on the sensor surface. On the other hand, it has been discussed that the surface density of the loaded TMV particles is strongly influenced by the experimental conditions of the TMV adsorption and surface properties of the solid-state substrate used (see e.g., [47,48]). 

In this work, field-effect capacitive Ta_2_O_5_-gate EIS sensors have been applied for a label-free electrical detection of TMV adsorption as well as receptor immobilization on TMV particles. Ta_2_O_5_ has been selected as a gate-insulating material with regard to its excellent material properties such as high pH sensitivity and stability, low hysteresis, and high corrosion resistance in a wide pH range [49]. The impact of TMV concentration on the EIS sensor signal and density of TMV particles adsorbed onto the Ta_2_O_5_ gate surface has been systematically studied by means of field-effect measurements and scanning electron microscopy (SEM). In addition, the surface density of TMV particles loaded under different incubation times was characterized by SEM. Finally, the label-free detection of Pen immobilization onto the TMV particles is demonstrated via field-effect measurements.

## 2. Materials and Methods 

### 2.1. Fabrication of EIS Sensors

**Figure 1:**
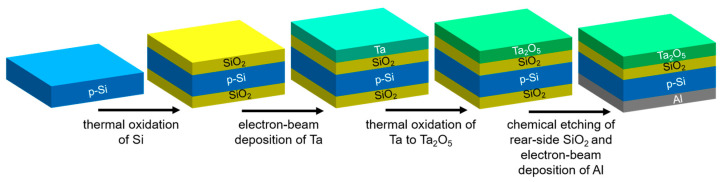
Fabrication process steps of the EIS chips (schematically).

The EIS sensor represents a bio/chemically-sensitive field-effect capacitor. The EIS chips consisting of an Al-p-Si-SiO_2_-Ta_2_O_5_ structure were fabricated from a ca. 380 µm thick Si wafer, with <100> orientation and 1–5 Ωcm specific resistivity. The fabrication process steps of the EIS chips are sketched in Figure 1. In its simplest embodiment, no photolithographic process steps are required. A thermally grown 30 nm thick high-quality SiO_2_ layer was formed by oxidation of the p-Si in a dry oxygen atmosphere at 1000 °C for about 30 min. This SiO_2_ layer guarantees the stable interface to the p-Si with low density of surface states. In the next step, a ~30 nm thick Ta film was deposited by electron-beam evaporation onto the SiO_2_ layer and oxidized in an oxygen atmosphere at 520 °C for 60 min, resulting in the formation of an approximately 60 nm thick Ta_2_O_5_ layer. The Ta_2_O_5_ serves as pH-sensitive transducer layer. The unwanted backside SiO_2_ layer, which is formed during the Si oxidation process, was removed by etching with hydrofluoric acid. Afterwards, an Al film (~300 nm thick) was deposited on the rear-side of the Si wafer by means of electron-beam evaporation to provide the electrical contact to the p-Si substrate, followed by tempering for 10 min in N_2_ atmosphere at 400 °C. Finally, the wafer was diced into 1 cm × 1 cm single chips. Before use, the chips were cleaned in an ultrasonic bath with acetone, isopropanol, ethanol and deionized water (each 5 min) and conditioned in buffer solution for at least 12 h.

### 2.2. Preparation of TMV Particles

**Figure 2:**
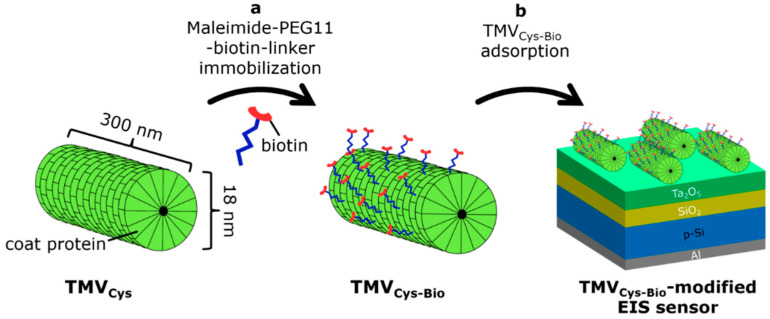
(**a**) Schematic of the biotinylation procedure of TMV_Cys_ particles and (**b**) loading of biotinylated TMV_Cys-Bio_ particles onto the EIS sensor surface.

In order to utilize TMV particles as bioreceptor nanocarriers for biosensing applications, they should be functionalized with linker molecules enabling an efficient immobilization of receptor molecules. In this work, we used genetically modified TMV particles with a cysteine residue (TMV_Cys_) at the amino terminus of each coat protein that provides a simple immobilization of bioreceptors via covalent binding of bifunctional biotin- and maleimide-terminated linkers to thiol groups of the cysteines, followed by affinity coupling of streptavidin-bioreceptor conjugates to the exposed biotin [50]. Figure 2 schematically illustrates the biotinylation procedure of TMV_Cys_ particles (a) and loading of biotinylated TMV_Cys-Bio_ particles onto the EIS sensor surface (b). The TMV_Cys_ particles, isolated from infected tobacco plants, were purified and biotinylated through covalent binding of bifunctional maleimide-polyethyleneglycol-biotin linkers (EZ-Link® Maleimide-PEG11-Biotin, ThermoScientific (Rockford, IL, USA)) to the thiol groups on the outer TMV_Cys_ surface. This results in TMV_Cys-Bio_ particles. The coupling efficiency of biotin linkers to the thiol groups on the outer TMV_Cys_ surface was determined densitometrically via image evaluation software ImageJ (Version 1.51k; Rasband, W.S., U.S. National Institutes of Health, Bethesda, Maryland, USA, https://imagej.nih.gov/ij/, 1997–2018) by using the procedure described in [30]. The large majority of accessible cysteine residues of TMV_Cys_ particles can be equipped with maleimide-polyethyleneglycol-biotin linkers, yielding an efficiency of biotinylation of approximately 94% and to about 2 × 10^3^ corresponding biotin sites per TMV_Cys-Bio_ particle, available for a high-density immobilization of streptavidin-bioreceptor conjugates. These results are in good agreement with data reported in [30,32]. The biotinylated TMV_Cys-Bio_ particles with a concentration of 5 µg/µL were stored in a 10 mM sodium-potassium-phosphate buffer (SPP, 10 mM NaH_2_PO_4_ and 10 mM K_2_HPO_4_), pH 7, at 4 °C. TMV_Cys-Bio_ solutions with different concentrations were prepared from the TMV stock solution of 5 µg/µL by dilution with 100 mM SPP buffer (pH 7, ionic strength 150 mM). For the details of TMV isolation, purification and biotinylation procedures, see [30,32]. To load the TMV_Cys-Bio_ particles on the EIS sensor surface, the Ta_2_O_5_-gate surface was incubated with the respective TMV solution.

### 2.3. Label-Free Electrical Detection of TMV Particles with EIS Sensors: Functioning Principle and Measurement Procedures

**Figure 3:**
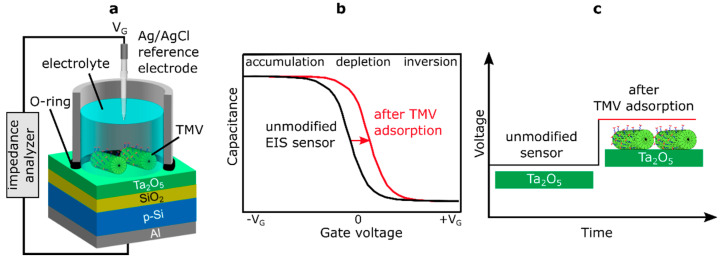
(**a**) Measurement setup of the TMV-modified EIS sensor chip, (**b**) typical shape of a high-frequency *C–V* curve for a p-type EIS sensor with characteristic regions of accumulation, depletion and inversion, and (**c**) ConCap response of the bare (unmodified) EIS sensor and after TMV adsorption.

For the characterization of EIS sensors, two basic operation modes are typically used: the capacitance-voltage (*C–V*) and the constant-capacitance (ConCap) mode [49,51]. Figure 3 shows the measurement setup of a TMV-modified EIS sensor chip (a), the high-frequency *C–V* curve for a p-type EIS sensor with the characteristic regions of accumulation, depletion and inversion (b), and the ConCap response (c) of a bare (unmodified) EIS sensor and an EIS sensor after TMV adsorption. 

Generally, for studying charge effects in a capacitive EIS sensor induced by adsorption of charged virus particles (such as TMV particles), the direction and amplitude of the *C*–*V* curve shift along the voltage axis in the depletion range is more relevant. Here, the total capacitance depends on the sign and amount of the charge of adsorbed virus particles, in addition to the applied gate voltage (*V*_G_). The isoelectric point (defined as pH where a particle carries no net electrical charge, pI) of native TMV particles lies at pH ~3.5 [19,22]. Hence, at pH values higher than pI, TMV particles are negatively charged. In case of a p-type EIS sensor, the adsorption of negatively charged TMV particles to the gate surface will diminish the width of the depletion layer in the Si and increase the overall capacitance of the field-effect sensor. This will result in a shift of the *C*–*V* curve to more positive (or less negative) gate voltages (Figure 3b). Both, the direction and the amplitude of gate-surface potential changes induced by the adsorption of charged TMV particles can be directly determined from the ConCap-mode response of the sensor, schematically shown in Figure 3c.

In this work, TMV loading onto the EIS sensors was studied in the dynamic ConCap mode by monitoring the temporal course of the sensor output signal, while the *C*–*V* mode was used to verify the correct functioning of the sensor as well as to choose the working point (i.e., constant-capacitance value) in the depletion region of the *C*–*V* curve for the ConCap measurements. The experiments on receptor immobilization on TMV particles were performed by applying both characterization modes.

For sensor operation in the *C*–*V* mode, a sweeping direct current (DC) gate voltage (*V_G_*) was applied between the reference electrode (RE) and the rear-side Al contact to set the working point. As reference electrode, an Ag/AgCl liquid-junction electrode has been applied (Metrohm, Filderstadt, Germany). A small alternating current (AC) voltage (20 mV) with a frequency of 120 Hz was superimposed to the DC voltage to measure the total capacitance of the field-effect sensor. In the ConCap mode, the capacitance at the working point of the field-effect sensor is kept constant by compensating surface potential changes (e.g., induced by the adsorption of charged virus particles) via applying an instantly sign-inverted voltage to the field-effect sensor using a feedback control circuit. In contrast to the *C*–*V* measurement mode, the ConCap mode allows real-time measurements as well as the study of drift effects and response time of the EIS sensor. For more details of *C−V* and ConCap modes, see, e.g., [49,51].

For the measurements, the EIS chip was installed in a home-made measurement cell (see Figure 3a) and sealed by a rubber O-ring. The contact area of the sensor surface with solution is defined by the inner diameter of the O-ring (8 mm) and was amounted to about 50 mm². The measurement cell with installed sensor chip was placed in a Faraday cage to prevent possible influences of ambient light and electromagnetic fields on the sensor signal. The measurements were performed at room temperature (RT) using an impedance analyzer (Zahner Zennium, Zahner Elektrik, Kronach, Germany). The pH value of all solutions used was controlled with a commercial pH-glass electrode (Mettler Toledo, Giessen, Germany). 

Before TMV-loading experiments, the leakage current between the RE and rear-side Al contact and the shape of the *C–V* curve of EIS chips were proven (in Titrisol^®^ buffer solution, pH 7). The aim of these experiments was the examination of correct functioning of the fabricated EIS sensors, evaluation of the intrinsic sensor parameters as well as the selection of appropriate chips for further surface modification steps. The leakage current was measured at an applied *V*_G_ ranging from −2.5 V to +2.5 V in 100 mV steps. Only chips having a maximum leakage current less than 10 nA and a typical shape of *C–V* curves with distinct accumulation, depletion and inversion ranges, respectively, were selected for further TMV-loading experiments. The mean pH sensitivity of bare Ta_2_O_5_-gate EIS sensors (*n* = 15) evaluated from the ConCap response was 58 mV/pH, which is in good agreement with previous results reported for Ta_2_O_5_ films [49]. 

For the loading of TMV particles, 50 µL TMV_Cys-Bio_ solution was dropped onto the sensor surface and incubated at room temperature in a humid chamber (the TMV_Cys-Bio_ concentration and the incubation time are specified in the respective sections). Afterwards, the sensor was washed three times with 1 mL 0.33 mM phosphate-buffered saline (PBS) buffer to remove unattached TMV particles, followed by the characterization of the TMV-modified EIS sensor with ConCap and/or *C–V* mode. After electrochemical characterization, the TMV-modified EIS sensors were removed from the measurement cell, rinsed with deionized water and prepared for SEM characterization.

### 2.4. SEM Characterization of TMV-Modified Chip Surface

In order to identify how the surface morphology of the Ta_2_O_5_-gate insulator was altered after adsorption of TMV_Cys-Bio_ particles, the surface of the EIS chips was characterized by SEM. SEM images were collected by a high-resolution Jeol JSM-7800F Schottky field-emission microscope (Fa. JEOL GmbH, Freising, Germany). A ~5 nm thin platinum-palladium layer was sputtered on the chip surface to increase conductivity. For each type of TMV-loading experiment, three chips were characterized by SEM method. The SEM images were taken from three representative spots on the chip surface and were used to calculate the surface density of adsorbed TMV_Cys-Bio_ particles. Due to the fact that the TMV_Cys-Bio_ particles can form long end-to-end multimeric structures or, contrarily, can contain smaller particle fractions (often 50–200 nm in length), not all adsorbed virus particles have the typical TMV length of 300 nm. Therefore, for calculating the surface density of TMV_Cys-Bio_ particles from SEM images, the amount of differently scaled particles was multiplied with appropriate correction factors.

### 2.5. Immobilization of the Enzyme Pen on TMV_Cys-Bio_ Particles on Pre-Loaded Chips

Pen was immobilized on the surface of TMV_Cys-Bio_ particles by bioaffinity binding of streptavidin-Pen (SA-Pen) conjugates to the biotin moieties. In previous experiments with TMV-assisted biosensors, this strategy has been applied successfully for a reproducible high-density immobilization of streptavidin-enzyme (e.g., glucose oxidase, glucose dehydrogenase, Pen) conjugates on TMV particles with full preservation of the enzyme activities in all cases; as a result, a better accessibility of the enzymes’ active centers for analyte molecules can be expected [25,31,32]. 

The enzyme Pen (from *Bacillus cereus*; specific activity: 2510 U/mg protein, Sigma–Aldrich, Darmstadt, Germany) was diluted to 1 mg protein/mL in 1×PBS. The SA-Pen conjugates were prepared using a commercial LYNX SA-antibody conjugation kit (LNK162STR, Bio-Rad, Feldkirchen, Germany). Before the immobilization of SA-Pen conjugates, the EIS chips were incubated with polymix buffer (for the composition of polymix buffer, see [52]) containing blocking agents (2% BSA) for 1 h at RT, to prevent possible unspecific adsorption of SA-Pen conjugates on the TMV-free areas of the sensor surface. For the immobilization of SA-Pen conjugates onto the surface of the TMV_Cys-Bio_, 50 μL SA-Pen solution (11.8 μg) was applied onto the TMV-equipped, BSA-treated EIS sensor surface and incubated for 2 h at RT in a humid chamber. For the details of the SA-Pen immobilization procedure, see [30,32].

## 3. Results

### 3.1. Impact of Virus Concentration on ConCap Signal and Surface Density of Adsorbed TMV Particles

The electrolyte-gated field-effect devices are known as very sensitive to charge (potential) changes at the gate insulator/electrolyte interface. Therefore, in principle, EIS sensors are able to detect the adsorption of charged macromolecules or nanoparticles onto the gate surface, since they are carrying an intrinsic molecular charge. This label-free detection principle has been demonstrated by different kinds of EIS sensors, e.g., for the label-free electrostatic detection of DNA immobilization and hybridization, biomarkers, polyelectrolytes, charged gold nanoparticles and carbon nanotubes [49,53,54,55,56]. In solution, virus particles are electrically charged. Thus, it can be suggested that adsorption of intact virus particles on the EIS surface will modulate the surface charge at the gate insulator, and consequently the total capacitance of the EIS structure, inducing a virus charge (concentration) -dependent sensor response.

**Figure 4:**
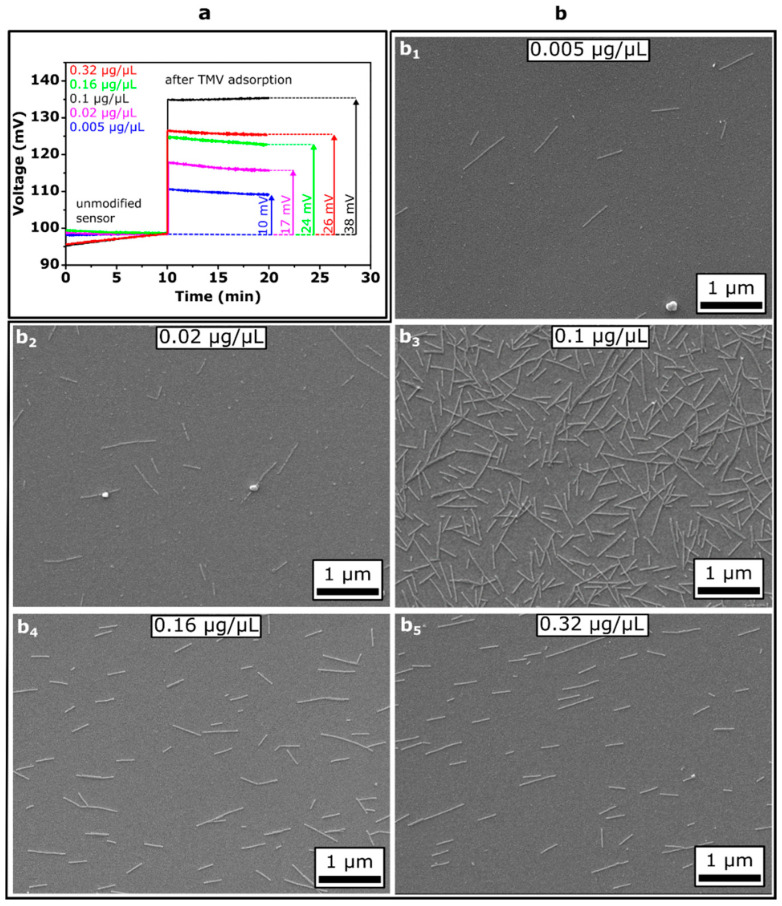
(**a**) ConCap responses of EIS sensors before and after loading of TMV_Cys-Bio_ particles from virus suspensions with different concentrations and (**b**) corresponding SEM images (**b**_1_–**b**_5_); incubation time for all experiments was 1 h.

First, we investigated the impact of the TMV_Cys-Bio_ concentration in solution on the TMV-modified Ta_2_O_5_-gate EIS sensor signal and the surface density of loaded TMV particles. For this, TMV_Cys-Bio_ suspensions of five distinct concentrations of 0.005, 0.02, 0.1, 0.16 and 0.32 µg/µL were applied in parallel onto separate sensor chips by drop-coating and incubated for 1 h. For each TMV_Cys-Bio_ concentration, three EIS chips were studied by means of ConCap mode measurements. Figure 4a shows ConCap responses of EIS sensors recorded in 0.33 mM PBS buffer (pH 8, ionic strength 5 mM) before and after loading of TMV particles from TMV_Cys-Bio_ suspensions with different concentrations. As can be seen, with increasing TMV loading concentration from 0.005 to 0.1 µg/µL, the signal change is increased from about 10 mV to 38 mV. However, surprisingly, after loading of TMV_Cys-Bio_ particles from the solution with concentrations of 0.16 and 0.32 µg/µL, the EIS signal change decreased again. It is important to note that the adsorbed assemblies of TMV_Cys-Bio_ particles on Ta_2_O_5_ surfaces withstood even multiple uses and treatment procedures (including rinsing and drying with nitrogen gas) or long-time storage. This feature was also demonstrated in our previous experiments, where TMV-modified Ta_2_O_5_-gate EIS penicillin sensors were stable over a time period of about one year without any significant loss of the sensitivity [32].

The results of the label-free detection of the adsorption of TMV_Cys-Bio_ particles with the capacitive field-effect sensors were supported by SEM surface characterization of the same sensor chips. Examples of SEM images of the sensor surfaces covered with TMV_Cys-Bio_ particles loaded from TMV solutions of different concentrations are presented in Figure 4b (b_1_–b_5_). In the SEM images, randomly oriented, laterally assembled single TMV particles with typical lengths of 300 nm, incomplete or partially disaggregated/broken short fragments of about 50–200 nm length as well as the formation of end-to-end aggregated long particles are clearly visible. The TMV_Cys-Bio_ particles did not form ordered structures on the Ta_2_O_5_ surface. A similar effect has been observed upon TMV adsorption, for instance, on bare [47] and oxidized [57] Si substrates, Pt electrodes [31] and Au-covered Si [28], and can be explained by the lack of the adsorbed particles’ mobility. When adsorption occurs, the respective TMV particles become “frozen” in position and orientation, resulting in randomly distributed particles with a large inter-particle spacing [47].

**Figure 5:**
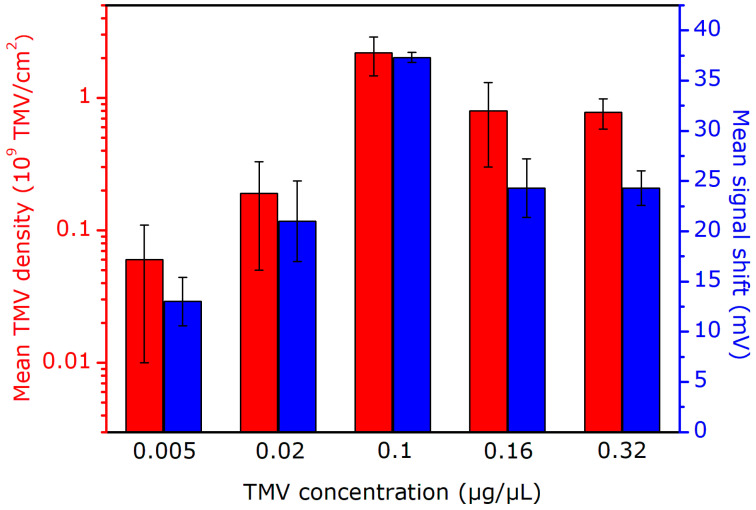
Mean surface density of TMV_Cys-Bio_ particles and mean amplitudes of ConCap signal changes for three sensors as a function of virus concentration in the solution. For each measurement three EIS chips were characterized (*n* = 3).

The mean surface density of TMV_Cys-Bio_ particles as a function of virus particle concentration in solution, evaluated from several SEM images for three prepared sensors, is shown in the bar chart diagram in Figure 5. Here, the mean amplitudes of ConCap signal changes for each three sensors as a function of TMV_Cys-Bio_ particle concentration are also presented. With increasing concentration of TMV_Cys-Bio_ solution from 0.005 to 0.1 µg/µL, the mean density of loaded virus particles on the Ta_2_O_5_ surface is increased from approximately 0.06 × 10^9^ to 2.2 × 10^9^ TMV/cm^2^. A similar trend in the amount or surface density of adsorbed TMV particles as a function of virus concentration in solution was observed upon loading of TMV_Cys-Bio_ particles on Pt electrodes [31] and TMV_Cys_ particles on a gold-covered Si wafer [58,59]. In addition, Bäcker et al. reported on saturation of the surface coverage of TMV particles on Pt electrodes by elevated virus concentration [31]. However, in our experiments, surprisingly, a further increase of virus concentration in the loading solution to 0.16 and 0.32 µg/µL resulted in a lowered density of TMV_Cys-Bio_ particles on the Ta_2_O_5_ surface of 0.8 × 10^9^ TMV/cm^2^ and 0.78 × 10^9^ TMV/cm^2^, respectively.

In the literature, different mechanisms of virus adsorption on various materials (e.g., Au, Pt, SiO_2_, mica, polyelectrolytes) have been discussed. These include, for instance, electrostatic and van der Waals interactions, hydrogen bonds to surface hydroxyl groups, hydrophobic and steric effects. The relative contribution of different interaction forces to adsorption and their dependence on the physicochemical properties of the viruses and substrates is rarely studied. To our best knowledge, the mechanism of TMV adsorption on Ta_2_O_5_ surfaces has not been analyzed so far. The experiments described above do not allow exactly explaining the observed decrease of the density of TMV_Cys-Bio_ particles on the Ta_2_O_5_ surface upon particle adsorption from a solution with high TMV concentration. One possible reason for this effect could be the formation of side-to-side aggregated TMV particles (TMV bundles) in the solution [47].

In further experiments, the TMV_Cys/Bio_ particles were loaded from the solution with a virus concentration of 0.1 µg/µL by which a maximum density of TMV_Cys/Bio_ particles on the Ta_2_O_5_ surface was observed, as well as the highest sensor signal in case of ConCap mode measurements.

The results presented in Figure 5 reveal a good correlation between the density of TMV_Cys-Bio_ particles on the EIS gate surface evaluated from the SEM images and the recorded ConCap signal changes. With increasing the mean density of TMV_Cys-Bio_ particles from 0.06 × 10^9^ to 2.2 × 10^9^ TMV/cm^2^, the mean amplitude of the signal change increased from 13 ± 2.4 to 37.3 ± 0.5 mV. Qualitatively, the observed signal behavior of the EIS sensor can be easily explained: the higher the surface density of adsorbed negatively charged TMV_Cys-Bio_ particles, the higher are the charge changes on the gate-insulator surface, yielding a larger sensor signal (see also Figure 4a). These findings demonstrate the ability of capacitive EIS sensors for label-free detection of intact TMV_Cys-Bio_ particles by means of their intrinsic molecular charge. In the future, field-effect EIS sensors could therefore be applied to quantify the amount (or density) of virus particles adsorbed on different materials without the use of a complicated and time-consuming SEM equipment, addressing a virus particle detector on field-effect basis. The major limitations of a label-free detection of virus particles by their charge with field-effect devices (including capacitive EIS sensors) are screening the virus charge by counter ions and/or possible non-specific adsorption of other charged species present in samples on the sensor surface. To overcome these limitations and thus to enhance the sensor performance, various strategies have been proposed recently. Examples are measurements in a low ionic-strength solution in order to reduce the influence of the counter-ion screening effect, or the use of agents to block non-specific adsorption, just to name a few (see, e.g., [43,49]).

### 3.2. Impact of Incubation Time on Loading of TMV Particles

**Figure 6:**
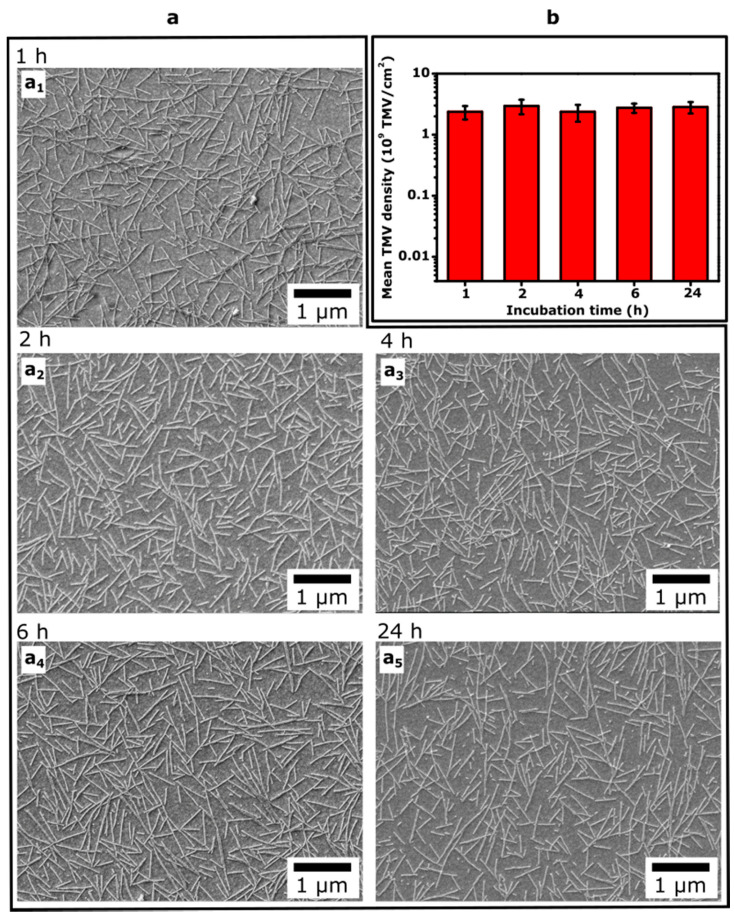
(**a**) SEM images taken from Ta_2_O_5_ surfaces after incubation of EIS chips with 0.1 µg/µL TMV solution for 1 h, 2 h, 4 h, 6 h, and 24 h (**a**_1_–**a**_5_); (**b**) Mean density of TMV particles on Ta_2_O_5_ surfaces evaluated from SEM images. For each incubation time, three EIS chips were characterized (*n* = 3).

In the literature, different incubation times have been reported for loading TMV particles onto various substrates (e.g., Au, Pt, mica, glass, SiO_2_, Ta_2_O_5_), ranging from about 20 min up to 18 h [28,31,32,47,58,59,60,61]. Therefore, we studied if the surface density of loaded TMV particles depends on the incubation time of Ta_2_O_5_-gate EIS chips with TMV solution. To this end, 50 µL of 0.1 µg/µL TMV_Cys-Bio_ solution was applied onto the EIS chips and incubated for 1 h, 2 h, 4 h, 6 h, and 24 h, respectively. Subsequently, the Ta_2_O_5_ surfaces with adsorbed TMV particles were characterized by SEM. The results of these experiments are summarized in Figure 6. The SEM images in Figure 6a (a_1_–a_5_) reveal that an increasing incubation time from 1 h to 24 h did not result in significant (or visible) differences of the TMV density on the Ta_2_O_5_ surface. The mean TMV density values evaluated from the SEM images for three sensors for each incubation time are overviewed in Figure 6b. An increase of the incubation time from 1 h to 24 h resulted in only small changes (~15%) of the surface density of TMV particles. For example, the mean surface density of TMV particles increased from 2.4 × 10^9^ TMV/cm^2^ to 2.8 × 10^9^ TMV/cm^2^ when varying the incubation time from 1 h to 24 h, respectively. Thus, it can be assumed that the TMV loading on the Ta_2_O_5_ surface is nearly completed within ~1 h incubation time. Therefore, in further experiments, the EIS chips were incubated with TMV solution for 1 h. 

### 3.3. Detection of Immobilization Event of Receptor Molecules onto the Virus Surface

As discussed in the Introduction section, functionalization of field-effect sensors with TMV particles as bioreceptor carriers can be used as a new platform to realize a large variety of biosensors. In order to demonstrate the ability of capacitive field-effect sensors for the label-free detection also of receptor immobilization onto the TMV particles, the enzyme Pen was used as a model receptor in this work. All surface modification steps of the EIS sensor, i.e., TMV_Cys-Bio_ loading, BSA adsorption and enzyme immobilization via affinity binding of SA-Pen conjugates to biotinylated TMV, were monitored by means of *C–V* and ConCap methods.

**Figure 7:**
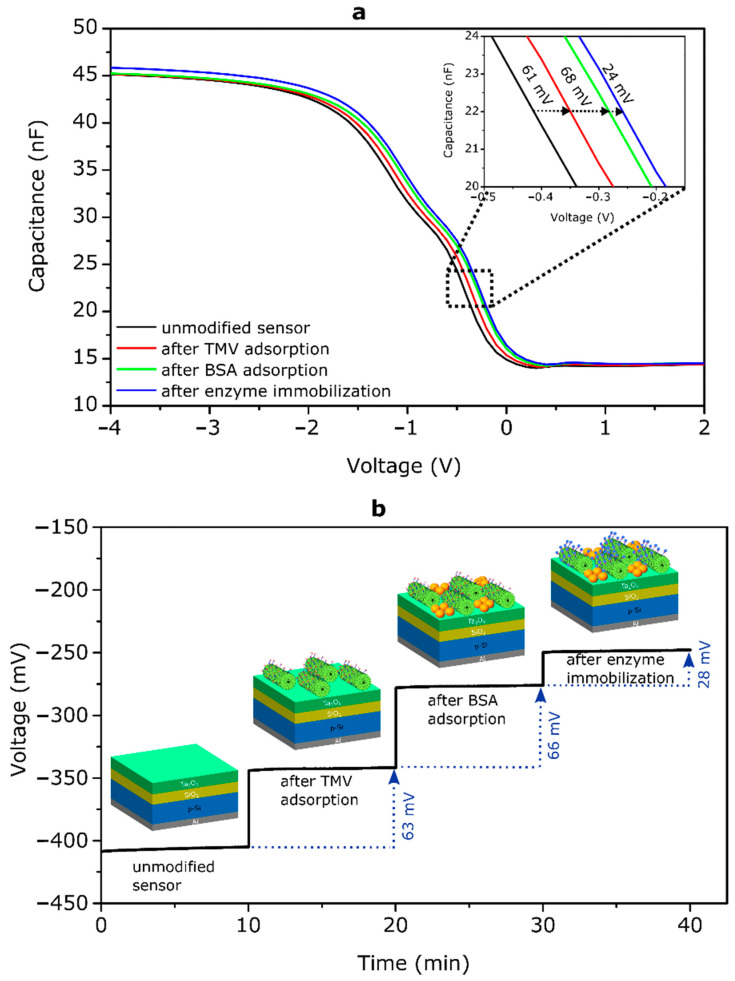
(**a**) *C–V* curves and (**b**) ConCap signals of an EIS sensor before and after each surface modification step (unmodified sensor, after TMV adsorption, after BSA adsorption, after enzyme immobilization).

Figure 7 depicts *C–V* curves (a) and ConCap signals (b) of an EIS sensor before and after each surface modification step recorded in 0.25 mM polymix buffer solution, pH 8. In this experiment, the TMV_Cys-Bio_ particles were loaded from 0.1 µg/µL TMV solution with an incubation time of 1 h. After the consecutive loading of TMV_Cys-Bio_ particles, adsorption of BSA molecules and Pen immobilization, the *C–V* curve of the bare EIS sensor shifted along the voltage axis for about 61 mV, 68 mV and 24 mV, respectively. The direction of the observed voltage shifts corresponds to a more negatively charged gate surface. The isoelectric points of TMV, BSA, SA and Pen reported in the literature amount to pI_TMV_~3.5 [19,22], pI_BSA_~4.8–5.4 [62,63,64], pI_SA_~5.6 [65] and pI_Pen_~5–6.4 [66,67], respectively. Therefore, in a measuring solution of pH 8 they are all negatively charged. Thus, the direction of the *C–V*-curve shifts correlates with the sign of charge of TMV_Cys-Bio_ particles, BSA molecules and SA-Pen conjugates. An adsorption of negatively charged particles/molecules decreases the width of the depletion layer in the Si, consequently increasing the space-charge capacitance of the semiconductor. As a result, the *C−V* curve will shift towards the direction of less negative (or more positive) gate voltages that in fact was observed in our experiments.

The ConCap signal of the same EIS sensor measured at a constant capacitance value of 22 nF is depicted in Figure 7b. The working point for the ConCap measurements was chosen from the depletion region of the *C−V* curves in Figure 7a. As can be seen, immediately after exposing the EIS sensor surface to the measurement solution, signal changes of about 63 mV, 66 mV and 28 mV were recorded after modification of the Ta_2_O_5_-gate EIS sensor surface with TMV_Cys-Bio_ particles, BSA blocking agents and enzyme immobilization onto the TMV surface, respectively. These signal changes correlate very well with the shifts of *C−V* curves along the voltage axis in Figure 7a. 

Qualitatively, the observed direction of the ConCap signal can be explained as follows: as it has been discussed above, modification of the EIS sensor surface with negatively charged TMV_Cys-Bio_ particles, BSA molecules and SA-Pen conjugates leads to space-charge changes in the depletion region in the Si and an increase of the total capacitance (at a constant gate voltage, see Figure 7a). To compensate these charge changes induced by the negatively charged species and keep the EIS capacitance in the ConCap mode constant, an instantly sign-inverted voltage has to be applied to the EIS sensor by means of a feedback circuit. Thus, the ConCap signals directly reflect the potential changes on the gate surface induced by the charged species. The ConCap signal amplitude depends on the density and the charge of TMV particles and BSA molecules as well as on the number and charge of immobilized SA-Pen conjugates per single TMV particle.

Finally, let us estimate the density of immobilized receptor (Pen) molecules per EIS sensor. As evaluated in Section 3.2, the mean surface density of TMV_Cys-Bio_ particles loaded from 0.1 µg/µL TMV solution with an incubation time of 1 h was about 2.4 × 10^9^ TMV/cm^2^. By assuming approximately 2 × 10^3^ biotin sites per TMV_Cys-Bio_ particle, available for the immobilization of SA-Pen conjugates (see Section 2.2) and a typical coupling efficiency of SA-enzyme conjugates to biotin sites of ~100% [25], the estimated density of enzyme Pen molecules amounted to 4.8 × 10^12^ Pen/cm^2^ or 2.4 × 10^12^ Pen molecules per EIS sensor with an active sensing area of 50 mm^2^. In fact, the density of immobilized receptor molecules will be somewhat smaller, because the portion of biotin sites on the TMV_Cys-Bio_ particle surface adsorbed to the sensor surface will not be accessible for receptor immobilization. 

An immobilization of enzymes on the TMV surface via bioaffinity binding of streptavidin-enzyme conjugates to biotin was shown to fully preserve the activity of a number of different enzymes, and a full accessibility of the active centers of these enzymes for analyte molecules [25,31]. For SA-Pen, respective in-depth analyses have been performed [30], and were extended to even demonstrate a long-term stabilization of the enzyme’s activity by TMV adapters on sensor chips for up to at least a year [32].

Taken together, the advantageous, high density display of target-specific enzymes on the surface of plant viral adapter particles make TMV-modified EIS sensors appealing alternatives to conventional layouts for many different biosensing applications.

## 4. Conclusions

Multivalent plant virus particles, in particular, TMV particles are very attractive nanoscaffolds for many fields of applications, including biochemical sensing, nano- and biotechnology. The TMV surface holds thousands of sites capable for a high-density and precisely positioned immobilization of various receptors, including enzymes. The characteristics of TMV-based biosensors, among others, depend on the density of TMV particles on the sensor surface, which in turn is influenced by the experimental conditions of TMV adsorption and surface properties of the sensor chip.

In this work, the influence of TMV_Cys-Bio_ concentration in solution (in the range of 0.005–0.32 µg/µL) on the surface density of TMV particles loaded to the Ta_2_O_5_ surface and the corresponding EIS sensor signal of the TMV-modified Ta_2_O_5_-gate was investigated by means of field-effect measurements and SEM. The observed ConCap signal changes correlate well with the density of TMV_Cys-Bio_ particles on the EIS gate surface as evaluated from SEM images. These results demonstrate the applicability of capacitive EIS sensors for label-free detection of intact TMV_Cys-Bio_ particles by their intrinsic charge. In addition, it was found that increasing the incubation time of the Ta_2_O_5_ surface with TMV solution from 1 h to 24 h did not result in significant differences of the surface density of the adsorbed TMV_Cys-Bio_ particles. Moreover, this newly developed capacitive EIS sensor was capable for the label-free detection of successful receptor immobilization onto the surface of the TMV_Cys-Bio_ particles via affinity binding of SA-Pen conjugates with the antibiotics-recognizing enzyme Pen as a model bioreceptor.

Even though in this work, the TMV_Cys-Bio_ particles were functionalized with Pen molecules as a model enzyme receptor, this new approach could most likely be adapted to other bioreceptors. The results achieved in this study underline a great potential of integrating plant viruses with field-effect devices as a novel platform in the field of label-free biosensing with numerous application possibilities. Future work will be directed to the development of theoretical models, which could describe the functioning of TMV-based field-effect biosensors taking into account the very complex character of the virus-modified gate surface/solution interface.

Plant viruses are among the major contributors to economic losses in agriculture with more than 50 billion EUR/year worldwide [68]. Therefore, there is a distinct interest in sensitive, fast and portable devices for an in-field or on-site early detection of plant pathogens/diseases [69,70]. On the other hand, the SARS-CoV-2 pandemic has dramatically highlighted the crucial role of virus detection technologies (especially with regard to rapid, accurate, reliable and easy-to-use, point-of-care detection systems) in the control of human infectious diseases. In this context, field-effect micro- and nanosensors could play an important role in point-of-care and on-site diagnostics of infectious diseases in the future.

## References

[1] Calò A., Eiben S., Okuda M., Bittner A.M. (2016). Nanoscale device architectures derived from biological assemblies: The case of tobacco mosaic virus and (apo)ferritin. Jpn. J. Appl. Phys..

[2] Culver J.N., Brown A.D., Zang F., Gnerlich M., Gerasopoulos K., Ghodssi R. (2015). Plant virus directed fabrication of nanoscale materials and devices. Virology.

[3] Fan X.Z., Pomerantseva E., Gnerlich M. (2013). *Tobacco mosaic virus*: A biological building block for micro/nano/biosystems. J. Vac. Sci. Technol..

[4] Love A.J., Makarov V., Yaminsky I., Kalinina N.O., Taliansky M.E. (2014). The use of *tobacco mosaic virus* and *cowpea mosaic virus* for the production of novel metal nanomaterials. Virology.

[5] Wen A.M., Steinmetz N.F. (2016). Design of virus-based nanomaterials for medicine, biotechnology, and energy. Chem. Soc. Rev..

[6] Zhang Y., Dong Y., Zhou J., Li X., Wang F. (2018). Application of plant viruses as a biotemplate for nanomaterial fabrication. Molecules.

[7] Mao C., Liu A., Cao B. (2009). Virus-based chemical and biological sensing. Angew. Chem. Int. Ed..

[8] Hwang I. (2014). Virus outbreaks in chemical and biological sensors. Sensors.

[9] Cardinale D., Carette N., Michon T. (2012). Virus scaffolds as enzyme nano-carriers. Trends Biotechnol..

[10] Narayanan K.B., Han S.S. (2017). Helical plant viral nanoparticles-bioinspired synthesis of nanomaterials and nanostructures. Bioinspiration Biomim..

[11] Van Kan-Davelaar H.E., van Hest J.C.M., Cornelissen J.J.L.M., Koay M.S.T. (2014). Using viruses as nanomedicines. Br. J. Pharm..

[12] Altintoprak K., Seidenstücker A., Welle A., Eiben S., Atanasova P., Stitz N., Plettl A., Bill J., Gliemann H., Jeske H. (2015). Peptide-equipped tobacco mosaic virus templates for selective and controllable biomineral deposition. Beilstein J. Nanotechnol..

[13] Koch C., Eber F.J., Azucena C., Förste A., Walheim S., Schimmel T., Bittner A.M., Jeske H., Gliemann H., Eiben S. (2016). Novel roles for well-known players: From *tobacco mosaic virus* pests to enzymatically active assemblies. Beilstein J. Nanotechnol..

[14] Wege C., Koch C. (2020). From stars to stripes: RNA-directed shaping of plant viral protein templates-structural synthetic virology for smart biohybrid nanostructures. Wires Nanomed. Nanobiotechnol..

[15] Koudelka K.J., Pitek A.S., Manchester M., Steinmetz N.F. (2015). Virus-based nanoparticles as versatile nanomachines. Annu. Rev. Virol..

[16] He J., Niu Z., Tangirala R., Wang J.-Y., Wei X., Kaur G., Wang Q., Jutz G., Böker A., Lee B. (2009). Self-assembly of *tobacco mosaic virus* at oil/water interfaces. Langmuir.

[17] Lomonossoff G.P., Wege C., Palukaitis P., Roossinck M.J. (2018). TMV particles: The Journey from Fundamental Studies to Biotechnology Applications. Advances in Virus Research.

[18] Sanctis S., Hoffmann R.C., Eiben S., Schneider J.J. (2015). Microwave assisted synthesis and characterisation of a zinc oxide/*tobacco mosaic virus* hybrid material: An active hybrid semiconductor in a field-effect transistor device. Beilstein J. Nanotechnol..

[19] Tiu B.D.B., Kernan D.L., Tiu S.B., Wen A.M., Zheng Y., Pokorski J.K., Advincula R.C., Steinmetz N.F. (2017). Electrostatic layer-by-layer construction of fibrous TMV biofilms. Nanoscale.

[20] Soto C.M., Ratna B.R. (2010). Virus hybrids as nanomaterials for biotechnology. Curr. Opin. Biotechnol..

[21] Capek I. (2015). Viral nanoparticles, noble metal decorated viruses and their nanoconjugates. Adv. Colloid Interface Sci..

[22] Alonso J.M., Gorzny M.Ł., Bittner A.M. (2013). The physics of *tobacco mosaic virus* and virus-based devices in biotechnology. Trends Biotechnol..

[23] Eiben S., Koch C., Altintoprak K., Southan A., Tovar G., Laschat S., Weiss I.M., Wege C. (2019). Plant virus-based materials for biomedical applications: Trends and prospects. Adv. Drug Deliv. Rev..

[24] Ge P., Zhou Z.H. (2011). Hydrogen-bonding networks and RNA bases revealed by cryo electron microscopy suggest a triggering mechanism for calcium switches. Proc. Natl. Acad. Sci. USA.

[25] Koch C., Wabbel K., Eber F.J., Krolla-Sidenstein P., Azucena C., Gliemann H., Eiben S., Geiger F., Wege C. (2015). Modified TMV particles as beneficial scaffolds to present sensor enzymes. Front. Plant. Sci..

[26] Koch C., Poghossian A., Wege C., Schöning M.J., Wege C., Lomonossoff G.P. (2018). TMV-based adapter templates for enhanced enzyme loading in biosensor applications. Virus-Derived Nanoparticles for Advanced Technologies.

[27] Zang F., Gerasopoulos K., Fan X.Z., Brown A.D., Culver J.N., Ghodssi R. (2014). An electrochemical sensor for selective TNT sensing based on *tobacco mosaic virus*-like particle binding agents. Chem. Commun..

[28] Fan X.Z., Naves L., Siwak N.P., Brown A., Culver J., Ghodssi R. (2015). Integration of genetically modified virus-like-particles with an optical resonator for selective bio-detection. Nanotechnology.

[29] Zang F., Gerasopoulos K., Brown A.D., Culver J.N., Ghodssi R. (2017). Capillary microfluidics-assembled virus-like particle bionanoreceptor interfaces for label-free biosensing. ACS Appl. Mater. Interfaces.

[30] Koch C., Poghossian A., Schöning M.J., Wege C. (2018). Penicillin detection by *tobacco*
*mosaic virus*-assisted colorimetric biosensors. Nanotheranostics.

[31] Bäcker M., Koch C., Geiger F., Eber F., Gliemann H., Poghossian A., Wege C., Schöning M.J. (2017). *Tobacco mosaic virus* as enzyme nanocarrier for electrochemical biosensors. Sens. Actuators B.

[32] Poghossian A., Jablonski M., Koch C., Bronder T.S., Rolka D., Wege C., Schöning M.J. (2018). Field-effect biosensor using virus particles as scaffolds for enzyme immobilization. Biosens. Bioelectron..

[33] Katz E., Poghossian A., Schöning M.J. (2017). Enzyme-based logic gates and circuits-analytical applications and interfacing with electronics. Anal. Bioanal. Chem..

[34] Poghossian A., Malzahn K., Abouzar M.H., Mehndiratta P., Katz E., Schöning M.J. (2011). Integration of biomolecular logic gates with field-effect transducers. Electrochim. Acta.

[35] Veigas B., Fortunato E., Baptista P.V. (2015). Field-effect sensors for nucleic acid detection: Recent advances and future perspectives. Sensors.

[36] Wu C., Bronder T., Poghossian A., Werner C.F., Schöning M.J. (2015). Label-free detection of DNA using a light-addressable potentiometric sensor modified with a positively charged polyelectrolyte layer. Nanoscale.

[37] Wu C., Poghossian A., Bronder T.S., Schöning M.J. (2016). Sensing of double-stranded DNA molecules by their intrinsic molecular charge using the light-addressable potentiometric sensor. Sens. Actuators B.

[38] Gao A., Chen S., Wang Y., Li T. (2018). Silicon nanowire field-effect-transistor-based biosensor for biomedical applications. Sens. Mater..

[39] Syu Y.-C., Hsu W.-E., Lin C.-T. (2018). Review - Field-effect transistor biosensing: Devices and clinical applications. ECS J. Solid State Sci. Technol..

[40] Poghossian A., Schöning M.J. (2014). Label-free sensing of biomolecules with field-effect devices for clinical applications. Electroanalysis.

[41] De Moraes A.C.M., Kubota L.T. (2016). Recent trends in field-effect transistors-based immunosensors. Chemosensors.

[42] Syedmoradi L., Ahmadi A., Norton M.L., Omidfar K. (2019). A review on nanomaterial-based field-effect transistor technology for biomarker detection. Mikrochim. Acta.

[43] Poghossian A., Jablonski M., Molinnus D., Wege C., Schöning M.J. (2020). Field-effect sensors for virus detection: From Ebola to SARS-CoV-2 and plant viral enhancers. Front. Plant. Sci..

[44] Ansari S.A., Husain Q. (2012). Potential applications of enzymes immobilized on/in nano materials: A review. Biotechnol. Adv..

[45] Poghossian A., Bäcker M., Mayer D., Schöning M.J. (2015). Gating capacitive field-effect sensors by the charge of nanoparticle/molecule hybrids. Nanoscale.

[46] Poghossian A., Schöning M.J., Schöning M.J., Poghossian A. (2018). Nanomaterial-Modified Capacitive Field-Effect Biosensors. Label-Free Biosensing: Advanced Materials, Devices and Applications.

[47] Yang L., Wang S., Fukuto M., Checco A., Niu Z., Wang Q. (2009). Structure and interaction in 2D assemblies of *tobacco mosaic viruses*. Soft Matter.

[48] Knez M., Sumser M.P., Bittner A.M., Wege C., Jeske H., Hoffmann D.M.P., Kuhnke K., Kern K. (2004). Binding the *tobacco mosaic virus* to inorganic surfaces. Langmuir.

[49] Poghossian A., Schöning M.J. (2020). Capacitive field-effect chemical sensors and biosensors: A status report. Sensors.

[50] Geiger F.C., Eber F.J., Eiben S., Mueller A., Jeske H., Spatz J.P., Wege C. (2013). TMV nanorods with programmed longitudinal domains of differently addressable coat proteins. Nanoscale.

[51] Klein M. (1990). Characterisation of ion-sensitive layer systems with a C(V) measurement method operating at constant capacitance. Sens. Actuators B.

[52] Yoshinobu T., Ecken H., Poghossian A., Lüth H., Iwasaki H., Schöning M.J. (2001). Alternative sensor materials for light-addressable potentiometric sensors. Sens. Actuators B.

[53] Bronder T.S., Poghossian A., Scheja S., Wu C., Keusgen M., Mewes D., Schöning M.J. (2015). DNA immobilization and hybridization detection by the intrinsic molecular charge using capacitive field-effect sensors modified with a charged weak polyelectrolyte layer. ACS Appl. Mater. Interfaces.

[54] Kumar N., Kumar S., Kumar J., Panda S. (2017). Investigation of mechanisms involved in the enhanced label free detection of prostate cancer biomarkers using field effect devices. J. Electrochem. Soc..

[55] Chand R., Han D., Neethirajan S., Kim Y.-S. (2017). Detection of protein kinase using an aptamer on a microchip integrated electrolyte-insulator-semiconductor sensor. Sens. Actuators B.

[56] Poghossian A., Weil M., Cherstvy A.G., Schöning M.J. (2013). Electrical monitoring of polyelectrolyte multilayer formation by means of capacitive field-effect devices. Anal. Bioanal. Chem..

[57] Lee B., Lo C.-T., Thiyagarajan P., Winans R.E., Li X., Niu Z., Wang Q. (2007). Effect of interfacial interaction on the cross-sectional morphology of *tobacco mosaic virus* using GISAXS. Langmuir.

[58] Manocchi A.K., Horelik N.E., Lee B., Yi H. (2010). Simple, readily controllable palladium nanoparticle formation on surface-assembled viral nanotemplates. Langmuir.

[59] Royston E., Ghosh A., Kofinas P., Harris M.T., Culver J.N. (2008). Self-assembly of virus-structured high surface area nanomaterials and their application as battery electrodes. Langmuir.

[60] Maeda H. (1997). An atomic force microscopy study for the assembly structures of *tobacco mosaic virus* and their size evaluation. Langmuir.

[61] Zang F., Chu S., Gerasopoulos K., Culver J.N., Ghodssi R. (2017). Biofabrication of *tobacco mosaic virus* nanoscaffolded supercapacitors via temporal capillary microfluidics. Nanotechnology.

[62] Wadu-Mesthrige K., Pati B., McClain W.M., Liu G.-Y. (1996). Disaggregation of *tobacco mosaic virus* by bovine serum albumin. Langmuir.

[63] Shi Q., Zhou Y., Sun Y. (2005). Influence of pH and ionic strength on the steric mass-action model parameters around the isoelectric point of protein. Biotechnol. Prog..

[64] Lee W.-K., Ko J.-S., Kim H.-M. (2002). Effect of electrostatic interaction on the adsorption of globular proteins on octacalcium phosphate crystal film. J. Colloid Interface Sci..

[65] Park J., Nguyen H.H., Woubit A., Kim M. (2014). Applications of field-effect transistor (FET)-type biosensors. App. Sci. Converg. Technol..

[66] Imanaka T., Oshihara W., Himeno T., Aiba S. (1983). Comparative studies on extracellular penicillinases of the same structural gene, penP, expressed in *Bacillus licheniformis* and *Bacillus subtilis*. Microbiology.

[67] Matthew M., Harris A.M. (1976). Identification of f3-lactamases by analytical isoelectric focusing: Correlation with bacterial taxonomy. J. Gen. Microbiol..

[68] Pallás V., Sánchez-Navarro J.A., James D. (2018). Recent advances on the multiplex molecular detection of plant viruses and viroids. Front. Microbiol..

[69] Cassedy A., Mullins E., O’Kennedy R. (2020). Sowing seeds for the future: The need for on-site plant diagnostics. Biotechnol. Adv..

[70] Khater M., de la Escosura-Muñiz A., Merkoçi A. (2017). Biosensors for plant pathogen detection. Biosens. Bioelectron..

